# Flame-Retardant Glass Fiber-Reinforced Epoxy Resins with Phosphorus-Containing Bio-Based Benzoxazines and Graphene

**DOI:** 10.3390/polym16162333

**Published:** 2024-08-18

**Authors:** Stanislav Trubachev, Alexander Paletsky, Egor Sosnin, Oleg Tuzhikov, Boris Buravov, Andrey Shmakov, Anatoliy Chernov, Ilya Kulikov, Albert Sagitov, Yuan Hu, Xin Wang

**Affiliations:** 1 Voevodsky Institute of Chemical Kinetics and Combustion, 630090 Novosibirsk, Russia; satrubachev@gmail.com (S.T.); e.sosnin@g.nsu.ru (E.S.); shmakov@kinetics.nsc.ru (A.S.); chernov@kinetics.nsc.ru (A.C.); ivkulikov@kinetics.nsc.ru (I.K.); sagitov@kinetics.nsc.ru (A.S.); 2 Department of Physics, Novosibirsk State University, 630090 Novosibirsk, Russia; 3 Chair of General and Inorganic Chemistry, Volgograd Technical State University, 400005 Volgograd, Russia; tuzhikovoleg@mail.ru (O.T.); byravov@ya.ru (B.B.); 4 State Key Laboratory of Fire Science, University of Science and Technology of China, Hefei 230026, China; yuanhu@ustc.edu.cn (Y.H.); wxcmx@ustc.edu.cn (X.W.)

**Keywords:** polymer composites, flame retardants, thermal degradation, mass spectrometry, glass fiber reinforcement, pyrolysis, flammability, epoxy resin, graphene, benzoxazine

## Abstract

This paper presents a study of the flammability and thermal decomposition products of glass fiber-reinforced epoxy resin (GFRER) with the addition of cardanol-based phosphorus-containing benzoxazine monomer (CBz) and graphene and their combinations in different proportions (up to 20 wt.%). The addition of CBz alone or in combination with graphene resulted in an increase in the limiting oxygen index (LOI) and self-extinguishing in the UL-94 HB test. The flame-retardant samples had better tensile mechanical properties than the sample without additives. The differential mass-spectrometric thermal analysis (DMSTA) of the thermal decomposition products of GFRER without additives and with the addition of CBz and graphene was carried out. CBz addition promoted the thermal decomposition of high-molecular-weight products of epoxy resin decomposition in the condensed phase and at the same time decreased the time of release of low-molecular-weight thermal decomposition products into the gas phase. Graphene addition resulted in an increase in the relative intensities of high-molecular-mass peaks compared to GFRER without additives.

## 1. Introduction

Laminates based on glass fiber and epoxy resin have high mechanical properties, dimensional and chemical stability, corrosion resistance, and low density; therefore, they are used in the transport industry (including aviation) and electrical engineering [1,2]. However, the flammability of epoxy resin is an important problem because the combustion of epoxy resin is accompanied by high heat release and the formation of toxic volatile products. The addition of various flame retardants based on metals [3], silicon [4,5], boron [6], carbon [7,8], nitrogen [9], and phosphorus [10,11] to epoxy resin can help solve this problem.

Benzoxazines are a relatively new class of heterocyclic compounds obtained by a Mannich-type condensation reaction. Benzoxazines are safe to use, have a long shelf life at room temperature, and do not release toxic volatiles during polymerization [12]. In addition, benzoxazine monomers can be synthesized using not only fossil fuels but also crops, plants, and their residues from which cardanol [13,14] and other natural phenol derivatives are obtained. Copolymerization reactions with benzoxazine in the presence of catalysts or under heating are accompanied by ring opening and lead to the formation of bonds with hydroxyl phenolic groups [15,16]. The materials obtained in these reactions may have better flame-retardant properties than materials without benzoxazines.

Grishchuk et al. [17] synthesized copolymers of epoxy resin and benzoxazine that obtained a V-1 rating in UL-94. Chen et al. [18] showed that the addition of bis(3-phenyl-3,4-dihydro-2H-benzo[e][1,3]oxazin-6-yl)methane to epoxy resin resulted in a 10.3 vol.% increase in LOI and a UL-94 V-0 rating. Adding phosphorus-containing benzoxazine to epoxy resin improves flame-retardant properties more significantly [19,20,21]. Gue et al. [22] synthesized the flame-retardant CBz based on the phosphorus-containing compound BA-DOPO, aniline, 1-(4-aminophenyl)ethenone and cardanol. The sample with the addition of CBz yielded a 32 vol.% LOI value compared to a 25 vol.% LOI value for the sample without the addition of CBz. Also, CBz addition resulted in a decrease in the peak heat release rate and the total heat release rate. A combination of CBz and graphene led to a further decrease in the flammability of epoxy resin.

Information about the volatile thermal decomposition products of polymer materials that serve as gas fuel in the oxidation reaction is necessary to understand the combustion mechanism. Mass-spectrometric studies of high- and low-molecular-weight products of thermal decomposition of epoxy resin have been conducted previously [23,24,25,26]. The objectives of this study were to establish the effect of CBz and graphene on the flammability and mechanical properties of GFRER and determine the thermal decomposition products of GFRER with the addition of CBz and graphene. The LOI, UL-94 HB, tensile stress tests, and DMSTA using a molecular-beam sampling system were conducted. The results of this study can be used to produce new reinforced materials for the needs of various industries and develop models for the thermal decomposition and diffusion combustion of reinforced materials based on epoxy resin with and without flame-retardant additives.

## 2. Materials and Methods

### 2.1. Materials

In this work, glass fiber-reinforced epoxy resin (GFRER) slabs 1.9–0.3 mm thick were studied. The slabs were prepared from prepreg based on T-10-14 fabric (surface density 290 g/cm^2^, fiber density 36 fibers/cm, and 8/3 satin weave) using vacuum molding. The content of the binder based on ED-20 epoxy resin and UP-606/2 curing agent (2,4,6-tris(dimethylaminomethyl)phenol) in the prepreg was ~35%. ED-20 has the following physical properties: content of epoxy groups 20 wt.%, viscosity 18 Pa·s, and molecular weight 400 g/mol. Graphene (RUSGRAPHENE, Moscow, Russia) and cardanol-based phosphorus-containing benzoxazine monomer (CBz) synthesized by the authors were used as flame retardants for epoxy resin. A detailed description of CBz preparation can be found in [22].

The phosphorus mass fraction in 1 g of CBz was ~0.0281. The flame retardants were mixed with epoxy resin to obtain six binder compositions (the detailed composition of the epoxy binder in mass fractions is given in Table 1). The maximum percentage of added graphene was 2 wt.%, and that of CBz was 20 wt.%.

### 2.2. Methods

All samples in Table 1 were investigated by the limiting oxygen index (LOI) [27] and UL-94 HB [28] tests. UL-94 HB is a horizontal burning (HB) test for the flammability of plastic materials for parts in devices and appliances. The accuracy of setting the oxygen concentration in the LOI test was ±0.2%. The tensile testing of the samples was conducted under normal conditions according to State Standard (GOST) R 56785-2015 (Analogue of ASTM D3039/D3039M-08) in the Research Complex of the Technology Support Center.

The mass peak intensity was measured using an automated mass-spectrometric complex based on a time-of-flight mass spectrometer with a molecular-beam probe sampling system [29]. The DMSTA method [30] was modified. A GFRER sample 5 + 30 mm^2^ in size without a backing plate was located in a reactor (quartz tube with an inner diameter of 10 mm). The preheated carrier gas flow (Ar or N_2_ depending on the measured mass peaks) was fed into the tube from below. Gas temperature was controlled with a type T thermocouple located at the top of the sample. Preliminary experiments showed that the visible aerosol particles of thermal decomposition products in the form of white smoke were formed when leaving the reactor at a distance of 2 cm from the reactor exit. Thus, probe sampling was performed before the condensation of pyrolysis products in the gas flow. A sonic quartz probe with an orifice diameter of 70 µm was heated to 250 °C. The time for measuring changes in the 24 mass peak intensities in the experiment was 0.16 s. The sample heating rate was ~150 K/min. The carrier gas flow rate under normal conditions was 2.5 cm^3^/s.

Preliminary experiments to determine the composition of thermal decomposition products of GFRER with and without flame retardants were performed using gas chromatography–mass spectrometry (GC-MS) (Agilent HP 6890/5973N, Santa Clara, CA, USA). Gas sampling for GC-MS was carried out using a syringe made of polypropylene and high-density polyethylene. Liquid products were preliminary condensed using nitrogen and were then dissolved in acetone. Species were identified using standard databases of mass spectra at ionizing electron energies of 70 eV [31].

## 3. Results and Discussion

### 3.1. Flammability Tests

The flammability test results (LOI, UL-94 HB) for GFRER samples are presented in Table 2. The changes in LOI for GFRER samples with flame retardants relative to the WA sample are shown in parentheses. The accuracy of the density determination for GFRER was ±100 kg/m^3^, and the accuracy of the LOI was ±1 vol.%.

All GFRER samples with flame retardants showed better results in flammability tests compared to the WA sample. Flame-retardant addition resulted in an increase in the LOI and a decrease in the flame spread rate or self-extinguishing in the UL-94 HB test. Moreover, adding CBz alone to GFRER was more beneficial for improving flame retardancy than adding a combination of CBz and graphene. The 10B sample had a higher LOI value than the 2G8B sample, and the 20B sample had a higher LOI than the 2G18B sample. The sample with 20 wt.% CBz had the highest LOI = 28.5%. Adding even 10 wt.% CBz provided self-extinguishing in UL-94 HB, whereas in GFRER samples with a combination of CBz and graphene, self-extinguishing was observed only in the 2G18B sample.

In a previous study, the addition of 6 wt.% graphene to similar GFRER samples with a close binder content (~35%) but of smaller thickness (0.95 mm) resulted in an increase in LOI from 22.4 to 23.9 vol.% (by 1.5 vol.%) [32]. In the present study, the addition of 2 wt.% graphene to GFRER resulted in a 2.1 vol.% increase in LOI. Thus, the relative flame-retardant effect of graphene in the GFRER composition weakly depends on its concentration, as was also noted previously [33].

### 3.2. Strength Characteristics

The tensile testing results are presented in Table 3. Flame-retardant addition resulted in an increase in the peak tensile load and elongation to fracture of the samples. The 2G18B sample showed the best results in this test. Epoxy resin with a combination of CBz and boron-doped graphene also showed the best results in tensile testing compared to samples with adding only CBz or only boron-doped graphene [22].

The flame retardants may improve the mechanical properties of GFRER for two reasons. The first is that CBz can increase the number of chains in the cured epoxy oligomer, which, in turn, increases the crosslinking density of GFRER samples. Second, these additives can increase the adhesion of epoxy binder to fiber glass, which also strengthens GFRER samples.

Graphene can improve the tensile strength of GFRER in the following way: When using graphene nanotubes and nanoplates, the dominant mechanism for increasing tensile strength is their pull-out and crack bridging [34]. When a crack opens, graphene pulls out of epoxy resin, resulting in energy dissipation by friction. This decreases the crack propagation rate if strong interfacial adhesion exists between graphene and epoxy resin.

### 3.3. Analysis of Pyrolysis Products by Mass Spectrometry (In Situ) and GC-MS

**Figure 1:**
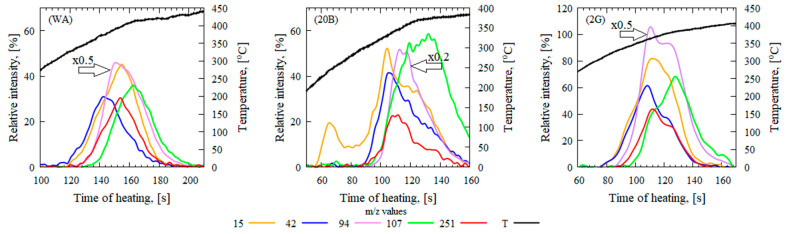
Mass peak intensity profiles at *m*/*z* = 15, 42, 94, 107, and 251 obtained by DMSTA for the WA, 20B, and 2G samples. The intensity at *m*/*z* = 94 is divided by 2 for the WA and 2G samples and by 5 for the 20B sample.

Figure 1 shows the characteristic mass peak intensity profiles of thermal decomposition products for GFRER without flame retardants and with the addition of CBz or graphene versus the gas carrier temperature obtained by in situ DMSTA. This experimental setup provides a complete picture of the thermal decomposition products, including easily condensing compounds.

The main objective of the analysis was to compare the sample without flame-retardant addition (WA) and with the addition of 20 wt.% CBz (20B) and 2 wt.% graphene (2G). Figure 1 shows that the thermal decomposition process can be formally divided into three (for the WA and 2G samples) or four (for the 20B sample) successive stages. Table 4 shows the maximum mass peak intensities (I_max_) normalized to the intensity of the carrier gas (N_2_). The contributions of the main aromatic decomposition products for GFRER were determined by using their unique mass-to-charge ratios (m/z), GC-MS, and the literature data [25,35,36,37]. The relative mass peak intensities of bisphenol-A and 2,2′,4,4′,6,6′-hexamethyl benzophenone were significantly decreased for the 20B sample. A decrease in the contribution of aromatic compounds with the addition of CBz to epoxy resin was also observed previously using FTIR [22]. At the same time, the 20B sample had a higher relative mass peak intensity of isopropyl phenol, p-isopropenyl phenol, and cresol. Hence, CBz in composition with epoxy resin can promote the thermal decomposition of aromatic products of epoxy resin decomposition in the condensed phase. Graphene addition resulted in an increase in the relative mass peak intensities of the above-mentioned compounds compared to the sample without additives. Due to the high specific surface area, graphene nanoplates can trap high-molecular-weight decomposition products of epoxy resin, which leads to their more complete thermal decomposition in the condensed phase.

**Figure 2:**
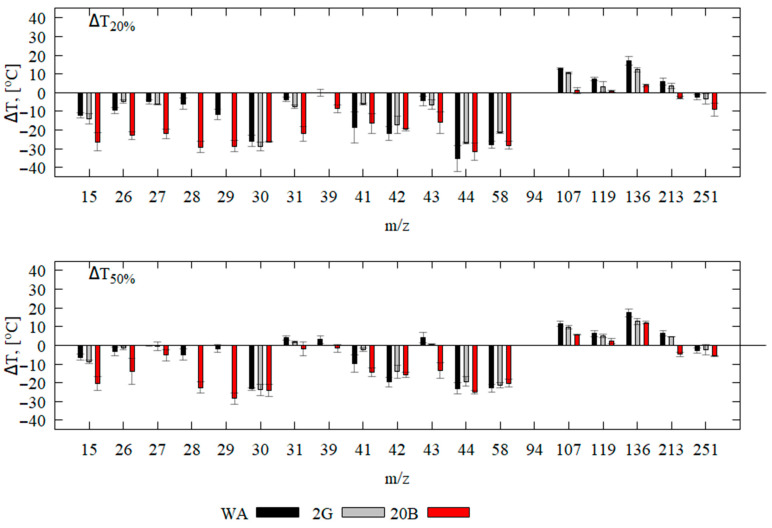
Release temperature of different thermal decomposition products relative to the release temperature of phenol for the WA, 2G, and 20B samples (at heights of 20% and 50% of their maximum value).

The release of gas-phase products of multi-stage thermal decomposition (the beginning of an increase in the corresponding mass peak intensities) starts at different temperatures. The species release time is usually determined with a high experimental error. Therefore, for subsequent signal processing, we used the mass peak intensities at heights of 20% and 50% of their maximum value at the corresponding temperatures (T_20%_(I*_m_*_/*z*_) and T_50%_(I*_m_*_/*z*_)). Since one of the main thermal decomposition products of epoxy resin according to the literature [25,26] is phenol, its mass peak was used as a reference peak for DMSTA against which the release time of other species was determined (earlier or later in temperature). Figure 2 graphically shows ΔT_20%_ = (T_20%_(I_94_) − T_20%_(I*_m_*_/*z*_)), ΔT_50%_ = (T_50%_(I_94_) − T_50%_(I*_m_*_/z_)) for different mass peak intensities. The negative values of ΔT_20%_ and ΔT_50%_ in Figure 2 indicate that the species were released into the gas phase earlier than phenol.

The addition of CBz significantly decreased ΔT20% and ΔT50% for low-molecular-weight species with mass peaks at *m*/*z* = 15–29 and 43, whereas the addition of graphene had little effect on these peaks. The release of low-molecular-weight thermal decomposition products occurred at a lower temperature for the 20B sample than for the WA sample. Thus, the heat release from low-molecular-weight products was not sufficient for the subsequent release of high-molecular-weight decomposition products (phenol and bisphenol-A) from the pyrolysis zone into the gas phase. The mass peak CH3+ (Figure 1 20B) also shows the earlier appearance of 20B decomposition products in the gas phase compared to other species, which supports the above explanation of the influence of CBz on the LOI. In the case of the sample without flame retardants, less energy for the thermal decomposition products is required for the onset of stationary combustion involving the release of high-molecular-weight (*m*/*z* ≥ 94) decomposition products into the gas phase.

## 4. Conclusions

The flame-retardant effect of the addition of cardanol-based phosphorus-containing benzoxazine monomer (CBz) (up to 20 wt.%) and graphene (up to 2 wt.%) and their combination on glass fiber-reinforced epoxy resin (GFRER) was investigated. The samples with the addition of CBz alone showed better flame-retardant properties in the LOI and UL-94 HB tests. The sample with a combination of 2 wt.% graphene and 18 wt.% CBz had the highest tensile test results. Thus, it can be concluded that CBz can be used to obtain materials with high flame retardancy requirements and the combination of CBz with graphene provides better mechanical properties of GFRER with a slight loss of fire-resistant properties.

The analysis of the thermal decomposition product of GFRER showed that CBz can promote the decomposition of bisphenol-A and other high-molecular-weight epoxy resin products to phenol in the condensed phase. At the same time, CBz resulted in an earlier release of low-molecular-weight decomposition products into the gas phase compared to phenol release, which may be responsible for the lower flammability of the samples with the addition of CBz. Graphene nanoplates can trap high-molecular-weight decomposition products of epoxy resin, which leads to their more complete thermal decomposition in the condensed phase.

## Figures and Tables

**Table 1:** Binder compositions of GFRER.

	Binder Content~35%					
	WA	2G18B	2G	10B	20B	2G8B
ED-20 Resin	100	100	100	100	100	100
UP-606/2	3	3	3	3	3	3
Graphene	-	2	2	-	-	2
CBz	-	18	-	10	20	8
Phosphorus, wt.%	0	0.41	0	0.25	0.46	0.2

**Table 2:** Flammability test results for GFRER.

FR (Flame-Retardant) Content, %	GFRER Sample	Binder Content, wt.%	Limiting Oxygen Index, vol.%	UL-94 HB (Rate of Flame Spread, mm/s)	Thickness, mm	Density, kg/m^3^
0%	WA	31 ± 2	22.7	HB (0.29 ± 0.03)	1.9 ± 0.1	1550
2%	2G	35 ± 2	24.8 (+2.1)	HB (0.30 ± 0.03)	1.8 ± 0.1	1650
10%	2G8B	34 ± 2	25.2 (+2.5)	HB (0.24 ± 0.02)	2.1 ± 0.1	1460
	10B	37.5 ± 2	25.7 (+3.0)	HB *	1.9 ± 0.1	1650
20%	2G18B	32.5 ± 3	26.1 (+3.4)	HB *	2.2 ± 0.1	1500
	20B	28 ± 3	28.5 (+5.4)	HB *	1.7 ± 0.1	1500

* Self-extinguishing.

**Table 3:** Tensile testing results for GFRER.

FR Concentration, %	Sample	Peak Load, kN	Displacement, mm
0	WA	11.7 ± 0.4	1.8 ± 0.1
2	2G	13.8 ± 0.3 (+2.1)	2.4 ± 0.1
10	2G8B	13.8 ± 0.3 (+2.1)	2.52 ± 0.03
	10B	13.8 ± 0.2 (+2.1)	2.52 ± 0.02
20	2G18B	15.5 ± 0.3 (+3.8)	4 ± 0.3
	20B	14.1 ± 0.1 (+2.4)	2.6 ± 0.1

The numbers in parentheses are the changes in peak load compared to the WA sample.

**Table 4:** Maximum mass peak intensities normalized to the nitrogen mass peak intensity (*m/z* = 14) for the thermal decomposition products of the WA, 20B, and 2G samples.

Proposed Compounds	Detected Mass-to-Charge Ratio (*m*/*z*)	WA	20B	2G
		I_max_, %	I_max_, %	I_max_, %
2,2′,4,4′,6,6′-Hexamethylbenzophenone	251	34 ± 3	25 ± 2	47 ± 5
Bisphenol-A	213	55 ± 6	29 ± 3	56 ± 6
Isopropylphenol	136	20 ± 2	29 ± 3	32 ± 3
p-Isopropenylphenol	119	64 ± 7	82 ± 8	67 ± 7
Cresol	107	48 ± 6	61 ± 6	59 ± 5
Phenol	**94**	**121 ± 22**	**286 ± 14**	**180 ± 16**
Acetone	58	35 ± 2	19 ± 2	35 ± 2
CH_3_^+^	15	59 ± 9	58 ± 3	70 ± 6

## Data Availability

Not applicable.
